# Effective Factors for Optimizing Metallophthalocyanine-Based Optoelectronic Devices: Surface—Molecule Interactions

**DOI:** 10.3390/molecules30030471

**Published:** 2025-01-22

**Authors:** Sakineh Akbari Nia, Aleksandra Tomaszowska, Paulina Powroźnik, Maciej Krzywiecki

**Affiliations:** Institute of Physics—Centre for Science and Education, Silesian University of Technology, Konarskiego 22B, 44-100 Gliwice, Poland; sakineh.akbarinia@polsl.pl (S.A.N.); aleksandra.tomaszowska@polsl.pl (A.T.); paulina.powroznik@polsl.pl (P.P.)

**Keywords:** phthalocyanine, molecular adsorption, molecular orientation, interface interaction, metal—organic hybrid layer

## Abstract

As a promising structure for fabricating inorganic—organic-based optoelectronic devices, metal—metallophthalocyanine (MPc) hybrid layers are highly important to be considered. The efficient charge injection and transport across the metal/MPc interface are strictly dependent on the precise molecular orientation of the MPcs. Therefore, the efficiency of MPc-based optoelectronic devices strictly depends on the adsorption and orientation of the organic MPc on the inorganic metal substrate. The current review aims to explore the effect of the terminated atoms or surface atoms as an internal stimulus on molecular adsorption and orientation. Here, we investigate the adsorption of five different phthalocyanine molecules—free-based phthalocyanine (H_2_Pc), copper phthalocyanine (CuPc), iron phthalocyanine (FePc), cobalt phthalocyanine (CoPc), vanadyl phthalocyanine (VOPc)—on three metallic substrates: gold (Au), silver (Ag), and copper (Cu). This topic can guide new researchers to find out how molecular adsorbance and orientation determine the electronic structure by considering the surface–molecule interactions.

## 1. Introduction

Metal phthalocyanines are composed of four π-conjugated macrocyclic molecules, surrounding a central metal atom [1,2,3,4]. They possess unique properties, including strong absorption and emission [5,6,7,8,9], photostability [10,11], thermal stability [6,12], chemical stability [6,13,14], and versatility [5,15]. These properties make MPcs valuable materials for a wide range of optoelectronic devices such as organic light-emitting diodes, organic solar cells, lasers, sensors, and photodetectors [6].

To improve the performance of the optoelectronic devices, a comprehensive understanding of the substrate—organic interfacial properties is essential. Since they can influence the electronic and optical behavior of the hybrid structure. Key factors, such as molecular orientation, dipole alignment (face-up/down), and molecular ordering (disordered phase, well-ordered, and densely packed), determine the overall properties of the structure and, consequently, the optoelectronic device. For instance, molecular orientation—e.g., flat-laying domains and inclined islands—affects light–matter interaction and interfacial charge transfer. Moreover, molecular dipole orientation as either face-up or face-down can affect the charge transport and device stability. The strength of the interfacial interaction—van der Waals and electrostatic forces—can determine the molecular orientation and arrangement [16,17,18,19].

The orientation of MPc molecules relative to the substrate surface determines the overlap between their frontier orbitals (highest occupied molecular orbital or HOMO and lowest unoccupied molecular orbital or LUMO) and the substrate’s electronic states [20]. This overlap is crucial for efficient charge transfer from the MPc molecules to the substrate or vice versa. A strong adsorption bond between MPc molecules and the substrate can enhance charge injection by providing a stable interface for electron transfer [21]. However, an excessively strong bond can hinder charge transport by trapping charge carriers. The formation of a well-defined interface between the MPc molecules and the substrate can provide a suitable platform for efficient charge injection [22,23,24].

Thanks to advanced measurement equipment such as X-ray (ultraviolet) photoelectron spectroscopy XPS (UPS), scanning tunneling microscopy and spectroscopy (STM and STS), and X-ray absorption spectroscopy (XAS), it has been possible to observe and understand the electronic properties and geometric configuration of the metal—MPc hybrid structure. So far, the adsorption configuration of a wide variety of metallic substrates—MPcs has been studied under ultra-high vacuum conditions [13,20,25,26,27].

This review aims to consider the influence of surface atoms as internal stimuli on the adsorption and electronic properties of MPc molecules on metallic substrates. Here, we investigate the adsorption of five phthalocyanine molecules including H_2_Pc, CuPc, FePc, CoPc, and VOPc on three metallic substrates such as Au, Ag, and Cu.

This selection of MPcs enables a systematic exploration of the impact of the central metal atom on the interfacial properties. H_2_Pc, the free-base molecule, serves as a reference [28,29,30,31,32], allowing us to observe how the incorporation of different metals with various properties such as different electronegativity—Cu, Fe, Co, and VO—influences interfacial interactions. CuPc, a widely studied MPc, is included to provide a benchmark for comparison [6]. FePc and CoPc, with their unpaired electrons (partially filled d-orbitals), offer insights into the role of magnetic properties in metal–organic interfaces [25,30,33,34,35]. Finally, VOPc, with its unique electronic configuration, provides a diverse system for investigating the effects of different metal centers on the behavior of the metal–MPc interface [36,37]. By understanding these factors, we aim to provide insights into the mechanisms governing MPc—substrate interactions and their implications for the performance of optoelectronic devices based on MPcs.

## 2. Influential Factors on Molecular Orientation

Molecular orientation on the inorganic substrate is influenced by various factors: internal and external. Internal stimuli, including substrate properties such as crystal structure (crystallographic) orientation, terminated atoms, and roughness, are among the most effective parameters. They can manipulate the surface energy and create local potential wells, influencing molecular adsorption and orientation. External stimuli, such as thermal processes and post-deposition annealing, can enhance molecular mobility, allowing them to be posited in different orientations. It consequently changes the molecular packing and orbital overlapping, managing the electronic properties of the thin molecular layer [38,39,40,41].

### 2.1. Internal Stimuli: Substrate Properties

#### 2.1.1. Crystal Structure

Different crystal structures, such as simple cubic, hexagonal closed pack (hcp), and face center cubic (fcc) offer distinct atomic arrangements and surface topographies. They can affect the interaction between MPc molecules and the substrate surface [42].

As an example to highlight the atomic arrangements and surface topographies, Au (111) (√3 × 22) reconstruction is well considered and understood in which twenty-two bulk-like positions have been occupied by twenty-three Au atoms. Therefore, in large scale images prepared from the reconstructed Au (111) surface, a chevron-like bending is observable [29]. Hence, Au (111) has neither a perfectly fcc nor a hexagonal lattice. It is contracted along three different crystallographic close-packed directions: $\left[101\right]$, $\left[\bar{1}1\bar{0}\right]$, and $\left[0\bar{1}1\right]$ [29,43,44]. This means that the rearrangement of its surface atoms is an intrinsic property of the material itself. Compared to unreconstructed surfaces like Ag (111) and Cu (111), the surface of Au (111) has different energy levels at different locations because of its non-uniform surface. 

The non-uniform energy areas and the presence of local energy minima significantly influence how molecules adsorb to the surface. Molecules are more likely to bind at an area with the energy minima. Ag (111) and Cu (111) surfaces have a more uniform energy distribution, and molecules are less likely to have specific preferred adsorption sites. The unique adsorption behavior of Au (111) can have significant consequences for the arrangement and organization of molecules on the surface affecting various properties and processes, such as chemical reactions and surface-based technologies [45,46,47].

#### 2.1.2. Terminated Atoms

Different terminated atoms have a crucial role in MPc adsorption and can present different atomic arrangements and electronic properties, influencing the interaction with organic molecules [48].

Originated by the terminated atom on the surface, the strong substrate—molecule interaction proposes site-specific adsorption and results in long chains, islands, and highly ordered structures [49]. It additionally can manage the molecule configuration and degree of rotation. Regarding this matter, MPc materials have been largely investigated on coinage metal surfaces [50,51,52]. As a result, it turned out that the surface symmetry of the substrate determines the adsorption strength of the organic molecule on the metallic surfaces. For instance, the substrate—molecule interaction of Cu/FePc is more severe when the four-fold Cu (100) is applied than in the case in which the two-fold Cu (110) is utilized [53,54]. Au (111) termination presents a highly symmetric surface with a close-packed arrangement of gold atoms. It often exhibits strong van der Waals interactions with MPc molecules, leading to a flat-lying adsorption configuration [44,55]. Meanwhile, Au (110) termination offers a more corrugated surface with alternating rows of closely packed gold atoms. The surface topography can potentially lead to tilted or upright configurations [56]. Within this survey, we provide a collection of molecular configurations on metallic surfaces with different crystallographic.

#### 2.1.3. Substrate Roughness

**Figure 1:**
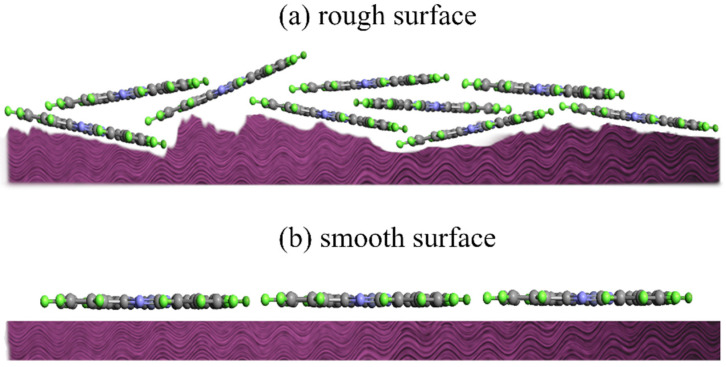
Schematic view of area provided by (**a**) rough surface, and (**b**) smooth surface.

The adsorption behavior, molecular distribution, and especially the appropriate thickness in which the MPc molecules start ordering on the metallic substrate surface are greatly affected by surface roughness. Rough surfaces provide more surface area, increasing the number of available adsorption sites for MPc molecules. It leads to higher overall adsorption and potentially denser molecular coverage, as schematically shown in Figure 1. However, the rough surfaces present a heterogeneous surface with different adsorption energies, resulting in non-uniform molecular adsorption and orientation, because, preferentially, MPc molecules are adsorbed by the sites with higher adsorption energies. Additionally, rough surfaces might favor tilted or upright orientations, compared to the flat-lying orientation often observed on smooth surfaces [57,58].

### 2.2. External Stimuli: Thermal Processes and Post-Deposition Annealing

The MPc molecular form ranges from amorphous to highly crystalline, including α and β as the most stable phases at room temperature. The phase of the MPc molecule can affect its arrangement in the crystal structure, which depends on the deposition method, rate, and temperature of the substrate [59]. Moreover, annealing of the deposited MPc films, as a common post-processing technique, influences the crystalline form, grain size, and surface roughness [60,61].

After depositing an MPc monolayer on metal surfaces by thermal evaporation technique, to produce a homogeneous organic layer on the metal substrate, it is recommended to anneal the MPc layer at temperatures close to the evaporation temperature [26,62,63]. Observed on the STM images, annealing the Ag (111)/FePc hybrid structure at 200 °C led to changes in topography and a more well-ordered molecular arrangement [64] because, at such a temperature, observing the chemical reactions, such as homocoupling and dehydrogenation, is possible. The physics of the substrate and the reaction process for a more reactive structure, Cu (111)/FePc, was considered as well to observe at what temperature the changes happen [26]. The gained results show that, after heating the Cu (111)/FePc hybrid structure, the symmetry reduction remains. Initially, the heating process (250 °C < annealing temperature < 300 °C) led to molecular layer reordering. However, at 320 °C, the desorption of the FePc molecule on the overlayer was observed. It also has been reported that the surface molecule binding was stronger and possessed a shorter distance. This is due to the rehybridization of the molecular states and the shift from π to σ surface interaction [26].

Beyond thermal processes, interfacial interactions are influenced by other environmental dynamic effects, such as humidity and the presence of atmospheric gases (oxygen and reactive species). Humidity can leave a profound impact. Water molecules can adsorb onto the substrate and organic layer altering the interfacial adhesion, degrading the organic film, and consequently affecting the longevity of the optoelectronic devices. Atmospheric gasses can interact with the interface causing oxidation and changes in the electronic properties of the hybrid layers [65,66]. These dynamic environmental factors need to be carefully considered and controlled to ensure the stability and reliability of organic electronic devices.

While a detailed discussion of these factors is beyond the scope of this review, it is crucial to acknowledge their significant influence on interfacial interaction.

## 3. MPc–Substrate Interaction: MPc Materials on Gold, Silver, and Copper Substrates

The substrate—organic interaction (such as Van der Waals, electrostatic, and covalent bonding) is the factor that can confine the MPc molecule in a specific adsorption site while the thermal energy is very low. Additionally, it is the main reason for the observed molecule configuration and influences the electronic properties since various adsorption configurations make different interactions between molecular-associated orbitals and the metal substrate affecting the charge transport properties. Regarding this matter, molecular adsorption configuration matters for the operation and efficiency of molecular-based devices. It is mainly considered by STM to observe the configuration, sites where a molecule has been adsorbed, and the symmetry break of the molecule (from four-fold to lower symmetries such as two-fold or, even, in some cases, one-fold) on the close-packed structures of the substrate [54,67,68].

There are some applications in which the MPcs have a common interface with the inorganic materials. For instance, in organic solar cell architecture, mainly one of the electrodes connected with the organic layer is metal. Therefore, different supporting substrates need to be considered and may have different effects on the molecule’s properties [69]. In this context, the interface between the metal substrates and the MPc molecules has been widely considered: both experimentally (different techniques) and theoretically (applying DFT methods). Au, Ag, and Cu are among the most popular substrates for MPc deposition due to their unique properties such as low reactivity and high conductivity, making them an ideal substrate for the long-term stability of MPc-based devices. Less chemically inert than Au, Ag is still a relatively stable substrate, especially when protected with a suitable coating. However, Cu is a more reactive substrate compared to Au and Ag; for this reason, it may show unpredictable electronic and optical behavior in the vicinity of organic molecules [70,71,72].

### 3.1. Free-Based Phthalocyanine (H_2_Pc)/(Au; Ag; Cu)

Evaluating the bonding configuration of H_2_Pc on Au revealed that reconstructed Au (111) with a surface including fcc—bridge—hcp regions [43,44] can affect directly the H_2_Pc molecule orientation and molecular crystallinity [29]. The H_2_Pc molecules at the low coverage are not adsorbed on the bridges; however, they are arranged in lines on the fcc and hcp regions. The number of the H_2_Pc molecules in both fcc and hcp regions are the same; however, theoretically and experimentally, it was reported that the fcc domain width (~1.9 nm) is almost ~1.3 fold bigger than hcp [43,44,47]. Therefore, the fcc domain is wide enough to adsorb the H_2_Pc molecules in all possible configurations but not wide enough to accommodate two H_2_Pc molecules, since the nearest neighbor distance for this molecule on the graphite substrate was reported as 1.3 nm. In the higher coverage, two different square-shaped clusters were observed for the Au (111)/H_2_Pc hybrid structure.

**Figure 2:**
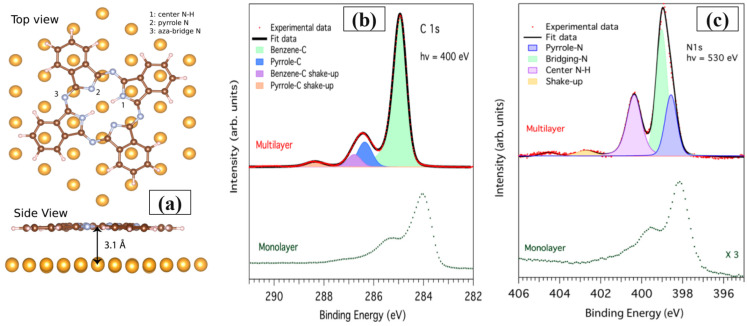
(**a**) Schematic view of the relaxed H_2_Pc molecule on the Au (111) surface: top view and side view. (**b**) C 1s and (**c**) N 1s core level spectra for coverage-dependent Au (111)/H_2_Pc hybrid structure. Reprinted (adapted) with permission from [31]. Copyright {2024} American Chemical Society.

Moreover, applying XPS, XAS, and DFT results, it was observed that, for multilayers of H_2_Pc on the reconstructed Au (111) (√3 × 22) substrate, molecules are still parallel to the surface, the same as submonolayers and monolayers (at room temperature) [31]. The top view and side view of the relaxed H_2_Pc molecule on the hexagonal Au (111), extracted from VASP calculation, is presented in Figure 2a [31] to confirm the flat-lay adsorption.

Provided by XPS analysis, C 1s spectra, Figure 2b, for both monolayer and multilayer of the organic molecule on the Au (111) substrate, confirms a shift in higher energy for the carbon_benezen energy line (0.8 eV) and the carbon_pyrrole energy line (1.0 eV). The reported energetic shifts are dedicated to the initial state effects and final state effects, which are the interaction mechanisms in the Au (111)/H_2_Pc interface, confirming the stronger interaction in lower coverage (monolayer) [31,73]. The obtained N 1s core level spectra for the Au (111)/H_2_Pc structure, presented in Figure 2c, show a shift to the higher binding energy as well. The changes in the energy lines of the components beneath the N 1s spectra (N-C and N-H) are the effects of the C 1s shift, which has also been observed in the other experimental and theoretical evaluations [31,74].

**Figure 3:**
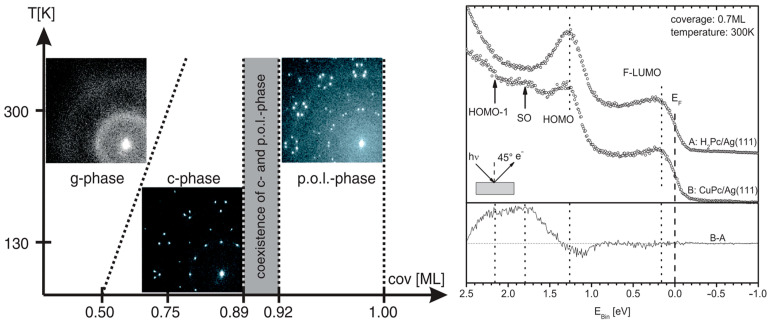
(**Left-hand side**) The SPA-LEED pattern of the different phases and (**right-hand side**) the UPS spectra of 0.7 ML of CuPc and H_2_Pc on Ag(111). Reprinted (abstract/excerpt/figure) with permission from [79]. Copyright (2024) by the American Physical Society.

On Ag (111), H_2_Pc molecules form a well-ordered commensurate phase, confirmed by normal incidence x-ray standing wavefield absorption (NIXSW) and spot profile analysis in low-energy electron diffraction (SPA-LEED). The interaction was investigated also by XPS measurements and is primarily driven by the nitrogen atoms in H_2_Pc, which approach the Ag (111) surface more closely than other atoms in the molecule. This leads to a slight bending of the phthalocyanine molecules, resulting in a strong chemisorptive bond between the nitrogen atoms and the substrate. UPS data indicate significant charge transfer from the silver to the H_2_Pc molecules, leading to a shift in both the HOMO and the formerly LUMO, consistent with the formation of chemisorbed states [75,76,77,78]. The SPA-LEED pattern of the different phases and the UPS spectra of 0.7 ML of CuPc and H_2_Pc on Ag (111) is shown in Figure 3.

**Figure 4:**
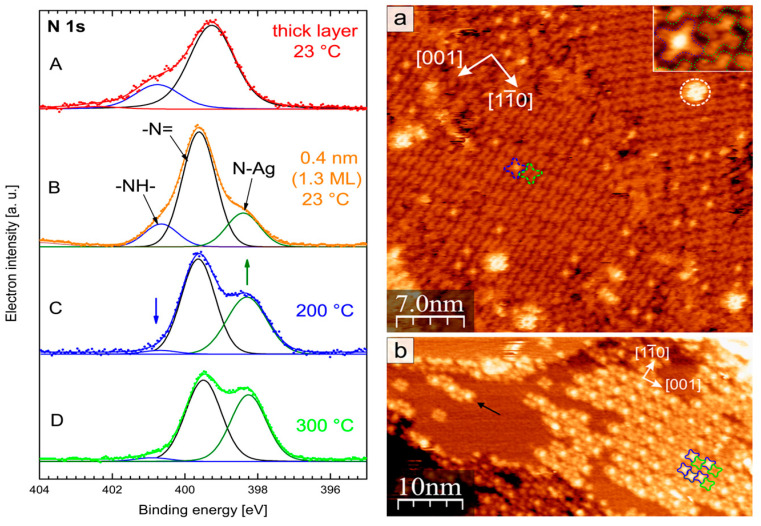
(**Left-hand side**) The thickness-dependent evolution of the N 1s core level spectra for Ag (110)/H_2_Pc [A = 7 nm, B–D = 0.4 nm] with the indicated temperature in the panels. (**Right-hand side**) The STM image for annealed Ag (110)/H_2_Pc: (**a**) H_2_Pc monolayer (100 °C), and (**b**) H_2_Pc monolayer (200 °C). Reprinted (adapted) with permission from [84]. Copyright {2024} American Chemical Society.

In contrast, the adsorption of H_2_Pc on Ag (110) leads to a self-metalation process, where silver atoms from the substrate are incorporated into the central cavity of the organic compound ring, forming silver phthalocyanine (AgPc). This self-metalation is more pronounced at step edges on the Ag (110) surface, where silver atoms are more mobile and can interact with the H_2_Pc molecules more readily. Studies using XPS and STM have confirmed the formation of AgPc on the Ag (110) surface, as presented in Figure 4, circled in white. The degree of metalation increases with the rising annealing temperature, a phenomenon attributed to the higher reactivity of Ag (110) compared to Ag (111) [80,81,82,83] where self-metalation does not occur [84].

**Figure 5:**
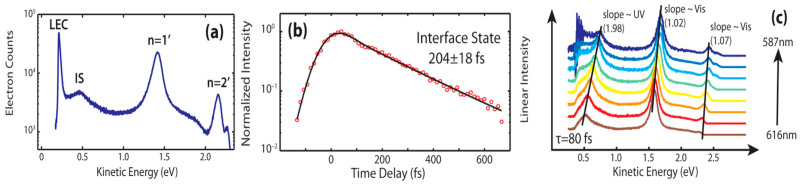
(**a**) The TPPE spectrum for H_2_Pc monolayer at τ = 0, (**b**) kinetics trace for the interface state, and (**c**) the TPPE spectra for the H_2_Pc monolayer at τ = 80 fs with fits, a sloop of one shows a visible. Reprinted (adapted) with permission from [78]. Copyright {2024} American Chemical Society.

Additionally, hybrid interface states are observed at the Ag (111)/H_2_Pc interface, as shown by time/angle-resolved two-photon photoemission (TPPE) studies, presented in Figure 5, which are located just above the Fermi level, arising from the interaction between the π-system of the H_2_Pc molecules and the Shockley surface state of the Ag (111) substrate. This formation modifies the electronic structure at the interface, contributing to the overall behavior of the system, such as strong dispersion, with an effective mass of ~0.5 m_e_, which is close to the Shockley surface state on clean Ag (111), 0.41 m_e_ [78,85,86,87].

In addition, a systematic consideration of mutual interaction between H_2_Pc and Cu (111) at room temperature revealed the adsorption configuration and electronic states of the H_2_Pc molecules [30,32,88,89]. The dI/dV curve obtained for Cu (111) with an fcc lattice, demonstrates a surface state peak at 200 meV, coming from the hybridization of s and p orbitals on this surface [88]. This peak indicates the existence of free-electron-like behavior in Cu (111) [88,90]. Defusing H_2_Pc molecules on the Cu (111) led to two-dimensional islands with a unit cell size of 1.5 × 1.5 nm^2^. The observed pattern is similar to that for Cu (111)/FePc [91] and Au (111)/CuPc [55]. In fact, it shows that the phthalocyanine-based molecule orientation is independent of the noble metal substrate when the noble metal includes an fcc (111) crystal structure, indicating a weak interaction between the phthalocyanine molecules and the noble metals [88].

The obtained local Density Of States (DOS) results from Cu (111)/H_2_Pc demonstrate that, for H_2_Pc molecular film, three peaks were observed at +600, +300, and −300 meV. It confirmed a metal-like behavior of the H_2_Pc on the Cu (111) surface, since the new peaks showed up close to the Fermi energy, leading to an increase in the local DOS [88,92].

**Figure 6:**
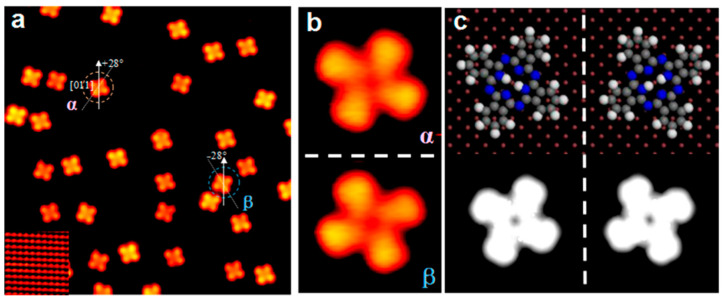
STM image gathered from the Cu (100)/H_2_Pc structure: (**a**) typical large area [V_b_ = −2.58 V; I_t_ = −0.05 nA], (**b**) two different H_2_Pc molecular orientations [V_b_ = −2.58 V; I_t_ = −0.04 nA], and (**c**) modeled top view of the Cu (100)/H_2_Pc structure and the corresponding adsorption configurations for H_2_Pc on the Cu (100) surface. Reprinted (adapted) with permission from [30]. Copyright {2024} American Chemical Society.

Diffusing H_2_Pc molecules on the Cu (100) surface showed random adsorption for the 0.35 ML coverage. The large area STM image revealed a four-folded adsorption for the H_2_Pc molecule on this surface with a depression site in the middle, offering a strong interaction in the Cu (100)/H_2_Pc interface, enough to present a thermal diffusion [30]. Similar to the ZnPc molecule deposited on the Cu (100) [93], H_2_Pc is adsorbed on the Cu (100) with two different azimuthal orientations relative to the [011] direction: ± 28°, as presented in Figure 6a,b and labeled by α and β. As presented in Figure 6c, the H_2_Pc molecule is centered on the hole site created by Cu atoms on the Cu (100) surface, which is the same as the Cu (100)/CuPc hybrid structure [30,94].

### 3.2. Copper Phthalocyanine (CuPc)/(Au; Ag; Cu)

The Au (110)/CuPc and the Au (110)/pentacene interfaces have been considered under the same condition to consider the effect of the Cu^2+^ central metal ion on the interaction between the organic molecule and Au (110) substrate [4]. The LEED patterns of these hybrid structures showed a (5 × 3) reconstruction for Au (110)/CuPc, which is different than what was detected for the pentacene case (6 × 3). For different organic and inorganic materials deposited on the Au surface, the ×3 periodicity was observed. Therefore, this periodicity is attributed to the Au surface arrangement [56,95,96]. While ×5 and ×6 periodicities are connected with the molecular dimension of the CuPc and pentacene. In this research, the high-resolution UPS results obtained from the organic coverage lower than 4 angstroms showed a strong similarity of the molecule-induced electronic structures. It demonstrates the crucial role of the π-delocalized orbitals in forming initial bonding with the Au substrate in both pentacene and CuPc cases [4].

Upon deposition of CuPc on Ag (111), it forms ordered molecular layers, where the molecules lay flat on the Ag (111) surface. This configuration is caused by the interaction between the π-conjugated system of phthalocyanine and the electronic states of the substrate. The adsorption process leads to a noticeable change, confirmed by UPS and XPS studies. Investigations reveal a shift in the HOMO and formerly LUMO (partially occupied due to charge transfer), which are indicative of strong chemisorptive bonding between the CuPc molecules and the Ag (111) surface. Further studies using SPA-LEED and NIXSW have demonstrated that CuPc forms a commensurate superstructure at sub-monolayer coverage on Ag (111). This ordered phase is stabilized by substrate-mediated interactions, which result in a continuous change in the lattice parameters as the phthalocyanine coverage increases. As a result of repulsive interaction, molecules show a substrate-induced long-range order that is crucial for understanding the adsorption mechanism [97,98,99,100,101,102].

For CuPc films deposited on silver, it was revealed that, at low coverage (below 2 nm), CuPc molecules interact more strongly with the silver surface than in bulk films attributed to enhanced absorption cross-sections at the interface, resulting from electromagnetic field localization near the silver surface [103]. However, in contrast to other metals such as potassium (K) or indium (In), silver does not strongly interact with CuPc to form new chemical bonds at the interface, making the Ag/CuPc interface largely inert and abrupt [104,105,106]. This lack of strong chemical interaction allows for a more predictable and controlled adsorption behavior, which is crucial for applications in optoelectronics, where precise control of the molecular arrangement and electronic states at the interface is necessary [107].

**Figure 7:**
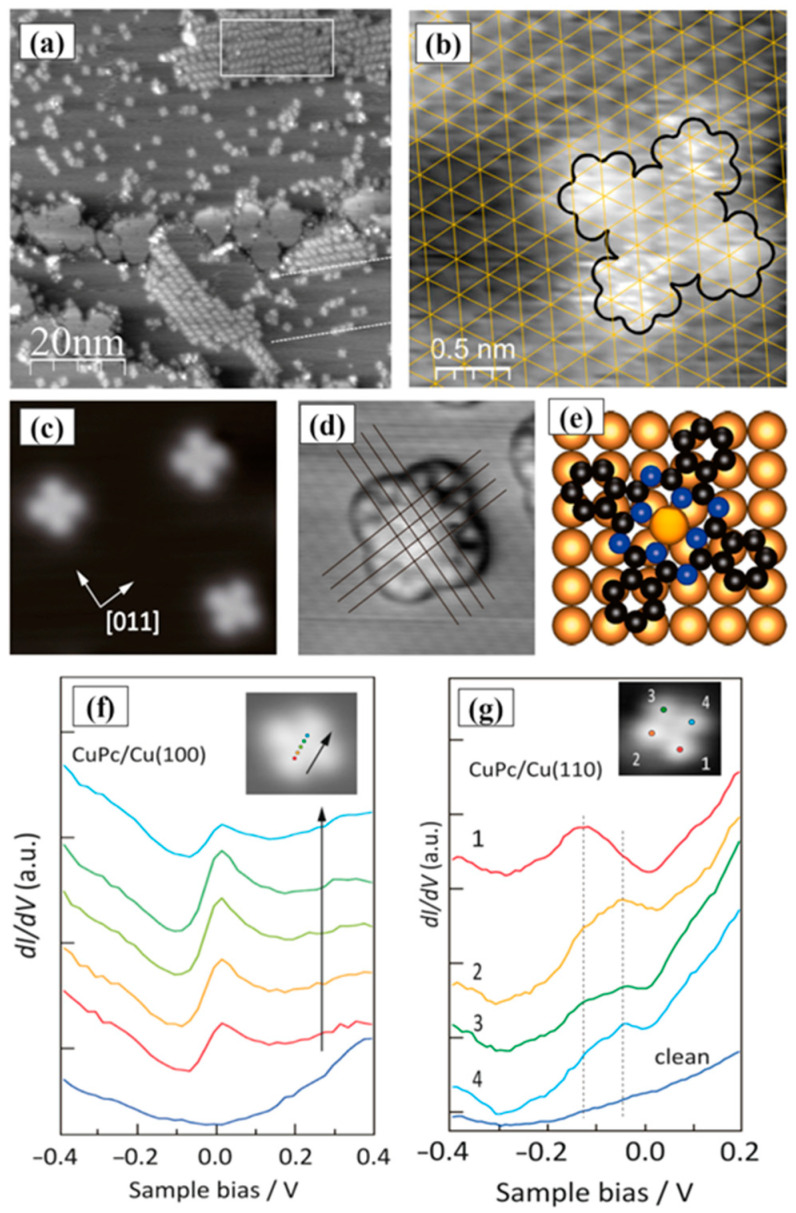
STM images of the (**a**) Cu (111)/CuPc hybrid layer [U_bias_ = 1.0 V; I_setpoint_ = 60 pA] and (**b**) 0.1 ML of CuPc deposited on the Cu (111) substrate [U_bias_ = +0.4 V; I_setpoint_ = 20 pA]. The hexagonal lattice of the Cu(111) is shown by yellow lines. Reprinted from [108], Copyright (2024), with permission from Elsevier. (**c**) STM images of CuPc molecules deposited on the Cu (100) [Vs = −0.1 V; I = 1 nA; size = 9.0 × 3.9 nm]; (**d**) CuPc molecules deposited on the Cu (100) utilizing no modified tip [Vs = −0.01 V; I = 5 nA; size = 3.9 × 3.9 nm]; and (**e**) the modeled view Cu (100)/CuPc hybrid structure. STS graphs for CuPc molecules on (**f**) Cu (100) and (**g**) Cu (110); the inset presents the points at which the spectra were collected. Reprinted from [94], Copyright (2024), with permission from Elsevier.

The interaction of the CuPc molecule with Cu substrate, including various crystal structures such as (111), (110), and (100), has been considered by different research groups [63,94,108]. Data analysis for Cu/CuPc interfaces clarifies the orientation of the CuPc on the copper substrates. Principally copper substrates possess six-fold symmetry while CuPc molecules show four-fold symmetry. When the CuPc molecule is adsorbed on less reactive metals such as gold, it keeps the four-fold symmetry; however, for the Cu (111)/CuPc case, the two-fold symmetry was observed demonstrating stronger interface (substrate-molecule) interaction [108,109]. When these molecules make chains or islands, it seems the molecule—molecule interaction is stronger than the substrate—molecule one since the symmetry reduction is still the same (two-fold) but in the opposite direction, indicating the domination of the molecule—molecule interaction on the substrate—molecule interaction (Figure 7a). The HR-STM image, presented in Figure 7b, confirmed that the CuPc molecule is centered on the copper atom in the case of the Cu (111) substrate [108]. However, for the Cu (100) substrate, the CuPc molecule is centered on the hole created by Cu atoms (Figure 7e) [94].

The electronic structure of Cu (100)/CuPc and Cu (110)/CuPc was evaluated utilizing STM and STS [94]. The STM images indicate that the organic molecule flatly lays on the Cu surface; however, the referenced symmetry for CuPc is different on these two substrates. The STM image of adsorbed CuPc molecules on the Cu (100) substrate is presented in Figure 7c,d. It shows that the four-fold organic CuPc molecules diffused on the Cu (100) surface and did not form an island even at higher thickness [94], suggesting a repulsive molecule—molecule interaction for the Cu (100)/CuPc interface. The obtained STM results from Cu (110)/CuPc show flat-laid dimmer CuPc molecules. Therefore, in contrast with Cu (100)/CuPc in Figure 7f, the STS results for Cu (110)/CuPc presented in Figure 7g showed a split LUMO, which was shifted below the Fermi level. It indicates that the hybridization of the d band has an essential role in aligning the energy levels of the Cu/CuPc interfaces.

### 3.3. Iron Phthalocyanine (FePc)/(Au; Ag; Cu)

**Figure 8:**
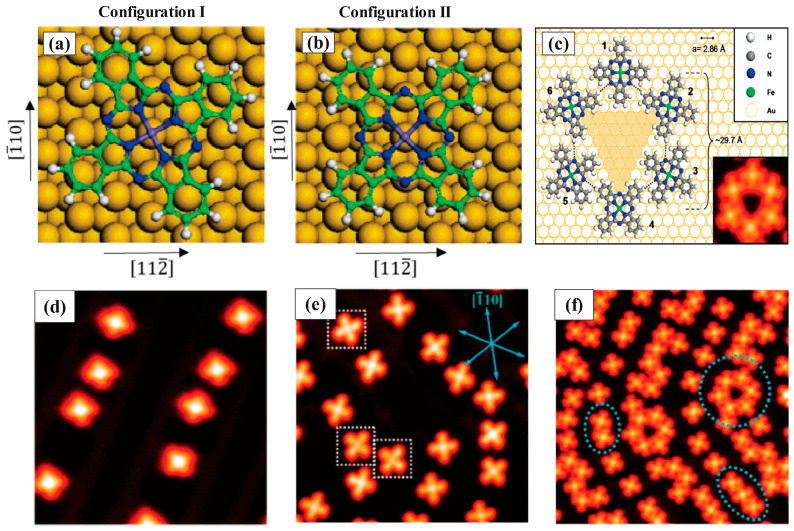
(**a**,**b**) Two preferred molecular orientations of FePc molecules on the Au (111). Both a and b orientations have been observed experimentally by the high-resolution STM technique. (**c**) Model of the FePc molecules’ hexamer formed on Au (111) [35]. STM images of (**d**) ~0.1 ML (14 nm × 14 nm), (**e**) ~0.3 ML (14 nm × 14 nm), and (**f**) ~0.6 ML (14 nm × 14 nm) FePc molecules on the Au(111) surface. Reprinted (adapted) with permission from [35]. Copyright {2024} American Chemical Society.

FePc molecular orientation on the Au (111) substrate has been comprehensively evaluated for different coverages, including low submonolayer, high submonolayer, and monolayer coverage by STM and DFT-based methods [35,42,110,111]. It seems that the orientation of the FePc on Au (111) depends on the coverage of the molecules on the substrate. Therefore, it represents both substrate—molecule and molecule—molecule interactions. For this hybrid structure, two flat-laying adsorption orientations were observed, adsorption configurations I and II, presented in Figure 8a,b. At the low submonolayer coverage, both orientations of the FePc molecule were observed. In this regime, FePc molecules prefer to be adsorbed on the Au (111) surface in an individual form since the surface—molecule interaction is stronger than molecule—molecule interaction (Figure 8d,e). Monomer, dimer, trimer, hexamers (Figure 8c shows the schematic view obtained by modeling), and short chains appear in high submonolayer coverage (Figure 8f) because, at the high coverage regime, the intermolecular distance decreases and the molecule—molecule interaction gets stronger. Finally, when the FePc molecules begin with ordering on the Au (111) surface and reach a monolayer structure, just the adsorption configuration I appears.

To manipulate the chemical properties of the FePc on Au, it is possible to attach various functional groups or different ligands to the central metal ion. As an example, the pyridine and ammonia electron donor ligands were attached to the iron ion [112,113]. Based on the obtained results from photoelectron spectroscopy (PES), at the interface (low coverage situation), the pyridine ligand does not affect the orientation of the FePc molecule. However, the coordination of ammonia led to significant changes in the electronic properties of the Au (111)/FePc hybrid structure due to the formation of covalent Fe—NH3 bonds, which significantly manage the FePc molecules’ magnetic properties [112].

**Figure 9:**
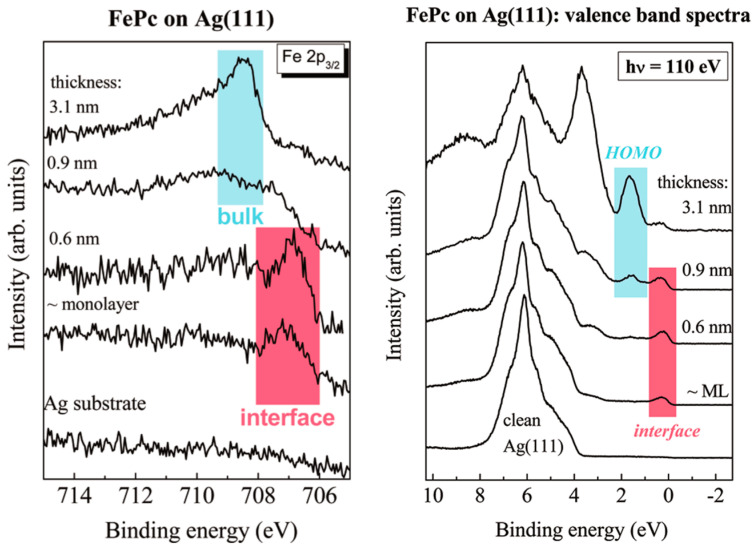
The thickness-dependent evolution of Fe 2p core and shallow HOMO level spectra. Reprinted (adapted) with permission from [120]. Copyright {2024} American Chemical Society.

Evaluation of FePc adsorption behavior showed a flat-laying molecule on the Ag (111) substrate [72,114,115]. The adsorption is driven primarily by the interaction between the Fe atom at the center of the phthalocyanine and the electronic states of the Ag (111) surface. This results in significant charge redistribution, which is confirmed by XPS and UPS data. These studies indicate the presence of hybridization between the Fe 3d orbitals and the substrate’s surface states, leading to the formation of new states at the interface [69,116,117,118,119]. This interaction stabilizes the molecular structure and is responsible for the observed shifts in the HOMO level and former LUMO of the FePc molecules, consistent with strong chemisorptive bonding [120]. Observed by TPPE spectroscopy, the hybrid states are located slightly above the Fermi level and play a crucial role in determining the electronic properties of the Ag (111)/FePc interface, contributing to enhanced charge transport and strongly influencing the overall electronic behavior of the system [78]. The evolution of the Fe 2p 3/2 and HOMO level for the Ag (111)/FePc structure are presented in Figure 9. The interface development has been marked by colored frames.

Additionally, XAS results revealed that the central Fe atom maintains a partial magnetic moment upon adsorption, although this magnetization is reduced compared to the bulk phase due to the interaction with the substrate. This quenching has been attributed to the hybridization of the Fe 3d orbitals with the surface states [120].

In contrast, on Ag (110), the surface allows for a different configuration for the FePc molecule where silver atoms may become more involved in the interaction with the Fe. However, there is no strong evidence for a significant metalation process like that observed for H_2_Pc on Ag (110). Also, the adsorption on Ag (110) is characterized by weaker molecule–substrate interactions [121,122,123] with FePc displaying less reactivity towards surface silver atoms compared to H_2_Pc.

**Figure 10:**
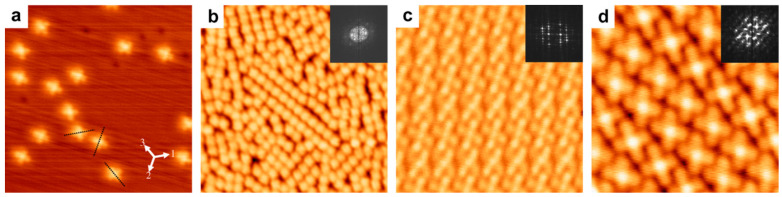
STM images of the Cu (111)/FePc hybrid layer for different overlayers: (**a**) At thickness much below 1 ML, FePc molecules appear isolated [V = 0.2 V; I = 100.0 pA; size = 23 × 23 nm^2^]. (**b**) At a larger stage, close but below 1 ML, short-range ordered islands showed up but without any preference (identified by the FFT results presented as inset) [V = 1.6 V; I = 100.0 pA; size = 30 × 30 nm^2^]. (**c**) [V = 0.29 V; I = 40.0 pA; size = 10 × 10 nm^2^] and (**d**) [V = 0.6 V; I = 30.0 pA; size = 7.7 × 7.7 nm^2^]) At thickness ~1 ML, the well-ordered domains are obvious. However, based on the FFT results presented as inset, there is no domain preference. Reprinted from [91]. Copyright (2024), with permission from Elsevier.

The surface—molecule interaction, molecule geometry, and the orientation of the FePc on the Cu surface have widely been explored both theoretically and experimentally [26,53,72,91,124]. At an early stage, for the Cu (111) surface with a hcp lattice structure and three-fold symmetry, the adsorption of the FePc molecule is perfectly aligned with the crystallographic axis of the Cu (111) since isolated FePc molecules are found, Figure 10a, in three equivalent orientations. However, for the thicker molecular layer, the molecular orientation is not significantly dependent on the surface properties [26,91], referring to a stronger substrate—molecule interaction than molecule—molecule interaction. Figure 10b–d show STM images for the Cu (111)/FePc hybrid layer for different molecular coverages [91].

A precise look in the STM images presented in Figure 10c,d indicates that domains were formed, and there is a mismatch angle of +14 and −14 degrees between the domains. Additionally, the unit cell size is 1.35 nm × 1.49 nm, but there is no preferred molecular orientation [26,91]. The symmetry reduction (two-fold symmetry) of the FePc organic molecule on Cu (111) was attributed to the formation of the anionic FePc and nonuniform charge distribution on the molecule axes [26,125,126]. The appearance of one-fold symmetry in sufficiently high coverage comes from the charge donation of the Cu (111) surface to the molecule and the out-of-plan distortion on the aromatic ring [26].

Since the lattice structure for Cu (100) and Cu (110) is four-fold and two-fold, respectively, the surface—molecule interaction in the Cu (100)/FePc interface is stronger than that in the Cu (110)/FePc structure [53]. The DFT calculation confirmed that the interaction resulted from hybridization between molecules’ π-orbitals (C 2p) and d-bands of the metal atoms (Fe 3d), leading to molecule bending and possible charge transfer [54]. Additionally, the obtained DOS results, in this research, confirmed the strong Cu (100)—FePc molecule interaction by releasing a predominant contribution to the valence level shift.

### 3.4. Cobalt Phthalocyanine (CoPc)/(Au; Ag; Cu)

Interface property consideration of the polycrystalline Au/CoPc and Au (100)/CoPc by photoexcited electron spectroscopies showed a relatively strong interaction between the Au substrate and the CoPc molecule. The XPS results showed a coupling between cobalt’s d orbital and s orbital of the Au substrate, which is similar to the observed behavior of the Fe central metal ion in the Ag (111)/FePc structure [34,127,128]. Similar results were reported in the case of Au (100)/FePc, depicting that the atomic structure of the Au substrate surface does not affect the interaction of the low-thickness (<4 nm) Au/CoPc hybrid structure [34]. After adsorbing CoPc on the Au (111) substrate, bias-dependent STM results demonstrated the quenching of the Co atom’s magnetic moment due to the substrate—molecule interaction, Au 6s and Co 3d_z_^2^ [34]. Recently, another study on the Au (111)/CoPc hybrid layer resulted in the optimization of the molecular ordering of this system by inserting hexa-peri-hexabenzocoronene (HBC) between substrate and organic molecules [129]. They observed that the CoPc imitates the crystal structure of the HBC interlayer and forms a hexagonal arrangement on Au (111)/HBC. Despite completely blocking charge transfer by the HBC layer, the interaction strength is almost the same for both Au (111)/CoPc and Au (111)/HBC/CoPc structures.

CoPc adsorption behavior on Ag (100) and Ag (111) distinct molecular arrangements is driven by interactions between the Co atom and the substrate [130,131,132,133,134,135]. On Ag (100), CoPc forms ordered molecular layers in a (5 × 5) R0 configuration, confirmed by STM and LEED studies [136,137]. Thermal desorption studies reveal that CoPc exhibits high thermal stability on Ag (100), with desorption and decomposition occurring simultaneously at temperatures exceeding 750 K. XPS and work function measurements indicate that the desorption energy ranges between 2.62 eV and 2.97 eV, suggesting strong interactions between CoPc and the silver substrate that enhance its thermal resistance. At elevated temperatures, structural transitions, such as the formation of a (√26 × √26) R11° phase, are observed, driven by annealing-induced molecular rearrangements [136].

**Figure 11:**
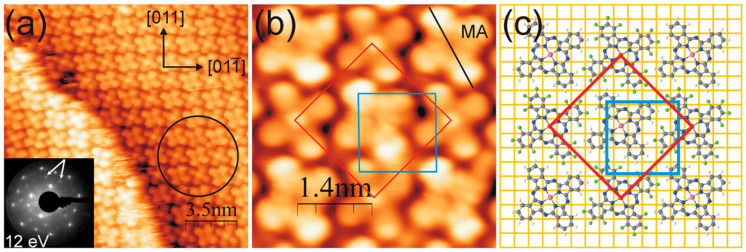
(**a**) STM image of CoPc mixed with F_16_CuPc on the Ag(100) [V = −0.78 V and I = 0.25 nA], with the LEED pattern as inset, (**b**) the HR-STM image of the structure [V = −2.36 V and I = 0.36 nA], and (**c**) the schematic model of the structure. Reprinted from [139], with the permission of AIP Publishing.

Additionally, DFT calculations suggest that the binding of CoPc to Ag (111) occurs via direct coordination of the Co with Ag atoms on the surface. The interaction primarily involves the d_z_^2^ orbital of the central atom, leading to strong chemisorptive bonding [138]. This contrasts with other metal phthalocyanines, such as CuPc, where the central metal atom does not participate in such localized charge transfer [139]. Combining CoPc with hexadecafluorinated copper-phthalocyanine (F_16_CuPc) on Ag (100), results in forming two molecular species with a (5√2 × 5√2) R45° phase. Where the CoPc molecules are arranged alternately with fluorinated phthalocyanine and the average distance between neighboring molecules is approximately 14.7 Å. This ordered arrangement is attributed to the interplay between direct molecule–molecule interactions and surface-mediated dipole–dipole interactions. Additionally, presented in Figure 11, the STM images reveal that the CoPc can be distinguished from F_16_CuPc based on their appearance, further supporting the idea that the molecular arrangement on Ag (100) is strongly dependent on the chemical composition of the adsorbed particles [139].

**Figure 12:**
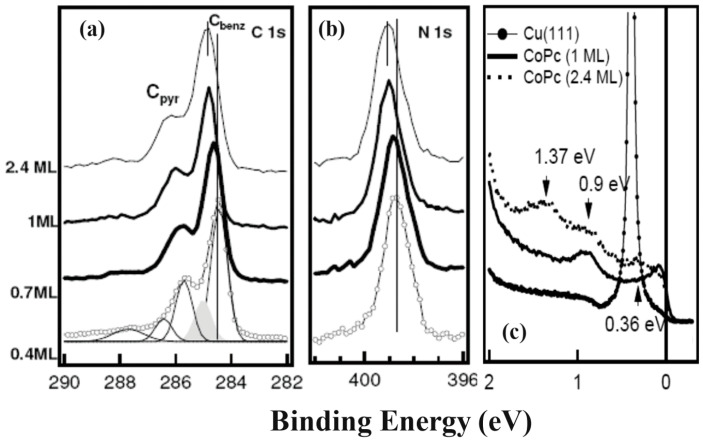
Thickness-dependent XPS and UPS results for Cu (111)/CoPc overlayers: (**a**) C 1s and (**b**) N 1s core level spectra. (**c**) Coverage-dependent valence band obtained for Cu (111)/CoPc. Reprinted (adapted) with permission from [25]. Copyright {2024} American Chemical Society.

Consideration of the geometrical arrangement of the CoPc as a paramagnetic molecule [25,140] on the Cu (111) and its electronic properties revealed deep insight into the Cu/CoPc interface [25,141]. Despite a relatively strong substrate—molecule interaction for the Cu/CoPc hybrid structure, the C 1s and N 1s core levels out of XPS measurements did not show any chemical bonding between C or N atoms and the substrate (Figure 12a,b).

One ML flat-laying CoPc molecule’s UPS measurements showed a shallow state at 0.9 eV, which is also present for the obtained spectrum of the 2.4 ML, Figure 12c. It has been attributed to the Cu—pyrrole/benzene interaction [25]. A similar feature was also revealed in the Au (110)/CoPc hybrid structure, but the peak was observed at 0.7 eV and responsible for the hybridization of π states localized at the N atom and the Au metallic states [142]. States like this disappear when the film thickness reaches several ML. Additionally, it was reported that there is a charge confinement in the Cu (111)/CoPc interface since the surface state of the metallic substrate, with the constant population, shifts toward the Fermi level; however, the electron effective mass grows [25].

### 3.5. Vanadyl Phthalocyanine (VOPc)/(Au; Ag; Cu)

In bulk structure, the VOPc molecule possesses a triclinic crystal structure with an overlap of π−π orbital, which increases charge transport. It makes the VOPc molecular layer a promising candidate for fabricating organic field effect transistors. VOPc is a nonpolar dipole molecule. Therefore, it is not only the molecular orientation but also dipole alignment and molecular ordering that can affect the inorganic—molecule hybrid structure [19,143,144].

**Figure 13:**
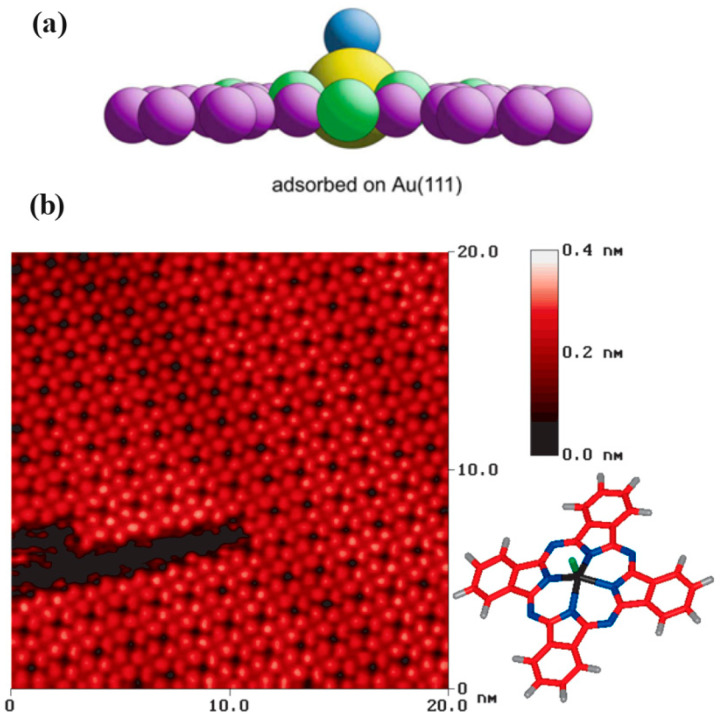
(**a**) The schematic of the VOPc adsorbed on the Au (111) substrate. Reprinted from [146]. Copyright (2024), with permission from Elsevier. (**b**) High-resolution STM image of the adsorbed VOPc molecule on the Au (111). Reprinted (adapted) with permission from [37]. Copyright {2024} American Chemical Society.

The VOPc molecule on the Au (111) has been considered in different studies [37,145]. Based on the results obtained by analyzing the high-resolution STM images, the unit cell is approximately square with a lattice spacing (unit cell vector) of 1.42 nm. In the center of the molecule, a depression region has appeared pointing to the oxygen atom of the VOPc molecule on top [146] since the O atom blocks conduction from the surface [37]. In both studies [37,145], it was pointed out that, for a monolayer of the VOPc on Au (111), the V = O bond of the VOPc molecule points out of the surface and the adsorbed molecule. Figure 13a,b show a schematic view of the orientation of the VOPc on the Au (111) and the obtained STM image presenting well-ordered VOPc molecules on the substrate.

In contrast, a disordered arrangement at the same coverage was observed for the VOPc molecule deposited on Ag (111), while the majority of the molecules were adsorbed with a V = O down, and the unit cell vector was grown to 2.12 nm [19]. However, the VOPc molecule keeps the four-fold symmetry with a repulsive intermolecular interaction. For higher coverage, not only the flat-laying but also the inclined molecular islands were observed. The ordered molecular layer was observed after the appearance of these islands comprising tilted VOPc molecules [19]. In both Ag (111)/SnPc as an interface with a nonplanar molecule and Ag (111)/CuPc, which is a structure including a planer molecule, the repulsive molecule—molecule interaction was observed. However, the origin of the repulsive intermolecular interaction was not reported as a dipole—dipole interaction, which is the reason for observing the repulsive interaction in the dipole molecules [63,147,148]. The origin of the repulsive interaction observed in this interface is attributed to the overlapping of the central metal atom orbital with the substrate orbitals and interfacial charge transfer [19,63].

**Figure 14:**
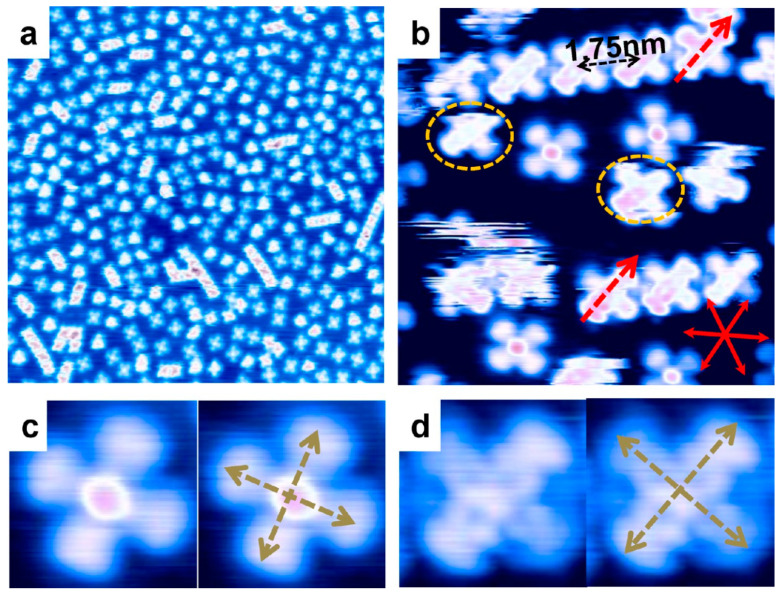
STM images of ~0.2 ML of VOPc molecules on the Cu (111) substrate: (**a**) Randomly distributed VOPc molecules and some face-down linear chains of VOPcs [V_tip_ = 1 V; I = 85 pA; size = 50 × 50 nm^2^]. (**b**) Face-down linear chains of VOPcs with the same orientation and 1.75 nm of molecular distance [V_tip_ = −0.1 V; I = 80 pA; size = 10 × 10 nm^2^]. Enlarged view [V_tip_ = 0.1 V; I = 80 pA] of (**c**) face-up and (**d**) face-down VOPc molecules. Reprinted (adapted) with permission from [19]. Copyright {2024} American Chemical Society.

Considered VOPc molecules on the Cu (111) surface with a thickness of 0.2 ML illustrated both face-up (oxygen atom upward) and face-down (oxygen atom downward) molecular configurations [19,149]. The face-up configuration with one-fold symmetry seemed more stable under the STM process than the two-fold symmetry face-down configuration as is obvious in the STM images presented in Figure 14. To become stable, face-down VOPc molecules aggregate in a linear chain with an intermolecular distance of 4√3 times the lattice of the Cu (111) substrate, which is almost 1.75 nm, as shown in Figure 14 [19]. It was pointed out that the symmetry reduction in the face-down VOPc molecule on the Cu (111) substrate can be dedicated to the degeneracy of the LUMO or π* since the VOPc molecule is well known for the double degeneracy of its LUMO [150]. In higher thicknesses, 1 ML coverage, the ordered molecular patterns appeared including face-down islands and mixed islands (face-up plus face-dawn islands, ~80% of the formed islands with an intermolecular distance of ~1.5 nm) [19].

## 4. Metal–MPc Hybrid Layer’s Applications, Challenges, and Limitations

So far, we have considered the crystallography of the metal substrate as a potential factor affecting the electronic structure of the MPcs. This insight is valuable for surface and interface engineering in electronic devices since the accurate molecule position and interface energy alignment with the supporting surface are essential for optimal device performance. Additionally, it can clear the challenges and limitations of applying the metal/MPc structure in device architecture, such as the non-uniformity of the organic MPcs on the metallic surface arising from the competition between substrate–molecule and molecule–molecule interactions.

Through this review, we mentioned that the crystallographic orientation of the substrate, particularly the termination atoms, plays a crucial role in determining the initial adsorption and subsequent orientation of MPc molecules. However, as the layer grows, intermolecular forces, such as π-π stacking and van der Waals interactions, can compete with substrate–molecule interactions, leading to the formation of islands or disordered regions. This non-uniformity can significantly impact the electronic and optical properties of the organic layer, ultimately affecting the performance of devices such as solar cells. To address this issue, precise control over the thickness of the MPc layer is essential. By carefully tuning the deposition conditions, it is possible to achieve uniform and well-ordered MPc films, which can enhance device performance and reproducibility.

As an example of the metal/MPc hybrid layer application, organic solar cells or organic photovoltaics (OPVs) are well known. MPcs are mainly applied in solar cells as hole transport materials (HTMs) to remarkably enhance the performance of the fabricated devices: in both positive–intrinsic–negative (p–i–n) and negative–intrinsic–positive (n–i–p) architectures [151]. The fill factor (FF), open circuit voltage (V_OC_), and power conversion efficiency (PCE) of the MPc-included OPVs can be tuned by controlling and tailoring the MPc layer (e.g., finding the optimized thickness and adding axial ligands) applied into the device structure [6].

Applying a dopant-free CuPc as an HTM in a mesoscopic perovskite solar cell on a Au electrode led to achieving 5.0% PCE [152]. Later on, by adding electron transport material (C60) into the structure, it even increased to ~15.5% [153] because a strong Coulomb interaction between the CuPc molecule and C60 leads to molecular rearrangement and thermal stability in the organic CuPc layer [154]. In this study [153], they also observed that the wide span of the light absorption of the CuPc (550–750 nm) affects the perovskite active layer’s absorption intensity and results in lower device efficiency. Furthermore, the CuPc thin film could not uniformly cover the distance between the metallic electrode and the perovskite layer, leading to a weak hole extraction. Thus, they tuned the hole extraction and the FF by optimizing CuPc thickness. To further increase the hole extraction, other research groups focused on the energy level alignment [155] and heating the substrate during the deposition [156].

As reported in a published paper [157], several MPcs, comprising CuPc, NiPc, CoPc, ZnPc, and H_2_Pc, alternatively were introduced as HTMs (~30 nm) to a solar cell structure: Au/MPc/Perovskite/Zinc Oxide/Fluorine Tin Oxide. Finally, the results showed that the Ni atom provides a higher electron contribution with the aromatic ring and led to 21.03% efficiency (certified) for the fabricated solar cell, which is a record [157]. It has also been reported that the NiPc thin layer has the potential to act as a surface passivator and enhance the crystallinity and uniformity of the perovskite layer, leading to higher device stability and performance [158].

To make it easy for readers, based on the study we are focusing on, the symmetry reduction and HOMO peak center (as one of the energy levels) has been summarized in Table 1.

## 5. Conclusions

Within the current review, we presented a survey of recent studies devoted to chosen metal—phthalocyanine interfaces. Further, we considered the influence of surface atoms as internal stimuli on the adsorption and electronic properties of phthalocyanine molecules on three different metallic substrates.

Growing the first monolayer of phthalocyanines with high quality on the substrate is one of their limitations. Thus, to control the growth process of the first monolayer, dominating the adsorption and orientation of the molecules on the substrate is essential. As an example of the metal—organic structures, the orientation of the five MPc molecules, including H_2_Pc, CuPc, FePc, CoPc, and VOPc, was reviewed on different metal substrates including Au, Ag, and Cu.

MPc molecules showed a flat-laying adsorption orientation at a low coverage regime with different configurations (different azimuthal angles) depending on the site where the MPc molecule is sitting, the rotation of the aromatic ring concerning the surface atoms, and the crystallographic structure of the substrate. However, in higher coverage (almost monolayer or higher), inclined molecules may appear. The observed molecular adsorption, orientation, ordering, and dipole alignment are attributed to the substrate—molecule interaction. Apparently, the Cu substrate provides more strength interactions with the MPc molecules as it is more reactive than Au and Ag. For these hybrid structures, the overlap of the substrate surface orbitals and the molecule orbitals was significantly affected by interfacial charge transfer.

While the phthalocyanine molecule possesses a four-fold symmetry in the gas phase, strong chemisorb metal—molecule interaction results in symmetry reduction. Moreover, disordered molecular ordering and orientation transition from flat-lay to inclined result from weak interface interactions and stronger molecule—molecule interactions.

From a longer perspective standpoint, for future research and to further advance our understanding of the inorganic—MPc interfaces, we suggest several promising directions: (1) considering the influence of defects and impurities of the substrate surface on the organic molecule adsorption and orientation, (2) exploring the effect of the external stimuli and dynamic factors such as thermal and environmental effects, and (3) applying other materials as a buffer layer between the substrate surfaces and MPcs to further organize the molecular layer.

To summarize, within the above survey, we presented technological conditions necessary to obtain particular adsorption regimes on metallic substrates. We do hope that the presented results will become significant support for scientists and engineers working on phthalocyanine—metal interfaces and will provide methodological hints for the investigation of similar systems in the future. That, in consequence, shall boost the organic-based technology making the perspective devices more efficient and affordable.

## Figures and Tables

**Table 1:** Symmetry reduction due to geometric and electronic effects and HOMO levels of different phthalocyanines adsorbed on metallic surface substrates. Note: although the H_2_Pc molecule has a two-fold symmetry in the gas phase, the MPCs exhibit four-fold symmetry [31].

Symmetry	H_2_Pc	CuPc	FePc	CoPc	VOPc
Au	Four-fold [159]	Four-fold [115]	-	-	Four-fold [46]
Ag	Two-fold [160]	Two-fold [161]	Four-fold [69]	Two-fold [134]	Four-fold [19]
Cu	Four-fold [40]	Four-fold and two-fold [102,116]	Two-fold and one-fold [26,131,132]	Two-fold [126]	One-fold and two-fold [19]
**HOMO (eV)**	**H_2_Pc**	**CuPc**	**FePc**	**CoPc**	**VOPc**
Au	1.6 [162]	0.71–0.82 [63]	1.4 [163]	1.25 (onset = 0.75) [34]	0.9 ^1^ [46]
Ag	1.5 [160]	1.25 [102]	1.4 [120]		1.2 ^2^ [46]
Cu	-	1.4–1.46 [63,141]	-	1.37–1.4 [25,141]	0.8 ^3^ [46]

^1^ The estimated value derived from Figure 3.16 in [46]. ^2^ The estimated value derived from Figure 3.21 in [46]. ^3^ The estimated value derived from Figure 3.33 in [46].

## Data Availability

Not applicable.
